# Implementation of food education in school environments improves pupils’ eating patterns and social participation in school dining

**DOI:** 10.1017/S1368980022002154

**Published:** 2022-12

**Authors:** Aija L Laitinen, Amma Antikainen, Santtu Mikkonen, Kaisa Kähkönen, Sanna Talvia, Silja Varjonen, Saila Paavola, Leila Karhunen, Tanja Tilles-Tirkkonen

**Affiliations:** 1 Department of Clinical Nutrition, Institute of Public Health and Clinical Nutrition, University of Eastern Finland, Yliopistonranta 1, Kuopio 70211, Finland; 2 Department of Applied Physics and Department of Environmental and Biological Sciences, University of Eastern Finland, Kuopio, Finland; 3 School of Applied Educational Science and Teacher Education, University of Eastern Finland, Joensuu, Finland; 4 Finnish Society for Food Education Ruukku, Helsinki, Finland

**Keywords:** Schools, Educational models, Food habits, Food education, Health promotion

## Abstract

**Objective:**

Schools can be an effective arena for food education. The Tasty School is a tailored teacher-driven food education model that provides tools for implementing food education in primary schools. This study aimed to investigate the effects of the Tasty School model on pupils’ eating patterns and experiences. We also aimed to assess the implementation strength of the Tasty School.

**Design:**

A quasi-experimental study was conducted during one school year 2019–2020 in fifteen intervention and ten control schools. The intervention schools implemented the Tasty School food education model. The pupils completed web-based baseline and follow-up questionnaires in class during a school day. The principals were interviewed after the intervention. The data were analysed using a mixed-effects model for repeated measures, accounting for the implementation strength and selected standardisation effects.

**Setting:**

A total of twenty-five general Finnish primary schools.

**Participants:**

1480 pupils from grades 3−6 (age 8–12 years) from five municipalities in Finland.

**Results:**

Percentages of pupils eating a balanced school meal increased in schools where food education was actively implemented (*P* = 0·027). In addition, pupils’ experience of social participation in school dining strengthened in schools where the Tasty School model was implemented (5-point scale mean from 2·41 to 2·61; *P* = 0·017).

**Conclusions:**

Healthy eating patterns can be promoted by the active implementation of food education in primary schools. The Tasty School model offers a promising tool for developing healthy eating patterns and increasing social participation among pupils not only in Finland, but also potentially in other countries as well.

Eating patterns are established during childhood^(1–4)^ and influence well-being into adulthood^(5–8)^. Despite the acknowledged importance of healthy eating patterns on well-being, eating patterns among Finnish school-aged children seldom fulfill the dietary recommendations^(9–12)^. Consumption of vegetables, whole grain products and products high in unsaturated fat is lower than recommended, whereas consumption of products high in sugar, salt and saturated fat is above recommended levels. In addition, children’s meal patterns are often irregular, and snacking is common^(13)^.

School is an excellent arena for promoting healthy eating patterns, as it reaches practically all children in Finland^(14,15)^. The Finnish national curriculum^(16)^ guides primary schools to set objectives and implement food education but does not offer any practical tools or model to support implementation. A previous study showed that the ‘Tools for Feeling Good’ Finnish food education model was effective in promoting regular meals, vegetable consumption and eating varied school lunches among fifth graders^(17)^.

A multitude of individual factors (e.g. age, genetics, body image), social and physical environments (e.g. parental feeding practices, peers, school) influence the eating patterns of school-aged children^(18)^. Children’s eating patterns and the prevalence of body dissatisfaction have been raised as an acute public health concern, and thus multicomponent health-promotion strategies are needed at various levels^(19)^. One promising approach is the Satter Eating Competence Model (ecSatter), which emphasises positive and flexible attitudes towards food and eating^(20)^. Eating competence has been found to be related to healthier eating patterns among children and adolescents^(21)^.

In Finland, a free hot meal is offered at school daily to all pupils from pre-school to upper secondary level. According to the recommendations of the Finnish National Nutrition Council^(22)^, a school meal should include a warm main dish, salad, fibre-rich bread with margarine and milk, buttermilk or a plant-based drink. Each pupil consumes approximately 2500 hot school lunches over their school years. A previous study demonstrated that eating a balanced school meal was associated with more regular meal patterns, the availability of healthier foods at home and an overall healthier diet^(13)^. However, only a small proportion (9 %) of pupils eat the recommended balanced school meal, although it is available for everyone^(23)^.

The ‘Tasty School’ model^(24)^ provides tools for implementing food education in primary schools and thus helps schools to meet the requirements of the School Meal Recommendations^(22)^ and Finnish National Curriculum^(16)^. The Tasty School model was developed in cooperation with primary schools and nutrition, food education and basic education experts by utilising a previous nutrition curriculum, Tools for Feeling Good, as a starting point^(17)^. The food education model extended the earlier curriculum to cover the entire primary school system (grades 1–6, age 7–12 years) and was diversified through a website that included an idea bank with learning materials, a self-assessment questionnaire and online training for teachers. The model was based on several theoretical approaches: Self-Determination Theory^(25)^, the Health at Every Size approach^(26)^, Eating Competence^(20)^, Sensory-Based Learning^(27)^ (i.e. Sapere), Mindful Eating^(28)^ and Intuitive Eating^(29)^. The Tasty School has a holistic approach as it integrates food education pedagogy in school subjects, school dining and the school environment. The model offers a wide set of tools for evaluating, planning and implementing food education in primary schools. At the pupils’ level, the aims of the Tasty School were promoting healthy eating patterns especially during school days, increasing positive attitudes towards school dining, and increasing social participation in food education in schools, as well as supporting positive body image and eating competence.

The aim of the current study was to investigate the effects of the Tasty School food education model on pupils’ eating patterns and experiences of school dining, eating competence and perception of body image. We also aimed to assess the implementation strength of the Tasty School.

## Methods

### Setting and participants

The present study had a quasi-experimental design^(30)^ with fifteen intervention schools, which were supported to implement food education based on a tailored, teacher-delivered Tasty School model and ten control schools that did not receive food education support^(24)^. The study was conducted during the school year 2019–2020 and included baseline and follow-up questionnaires addressed to all grades 3–6 pupils in participating schools. Pupils’ parents were informed about the study via the schools’ web interface tool. The participation of the pupils was voluntary, but none of the parents declined to approve their child‘s participation in the study. Additionally, all principals of the intervention and control schools were individually interviewed by telephone after the intervention in June or October 2020.

The recruitment process commenced in January 2019 by contacting municipalities’ directors of education and inviting them to participate in the study. Based on their previous cooperation, six municipalities were contacted, and five municipalities located in southern and eastern Finland participated in the study. The population of these municipalities ranged from 10 000 to 120 000 and eight-three primary schools were located across the five municipalities.

Recruitment of schools from participating municipalities was carried out in spring 2019 by the directors of education. The aim was to recruit fifteen intervention and fifteen control schools in keeping with the project’s resources. Due to the varied resources of schools, the participating schools were able to choose their status in the study, either as an intervention school or a control school. The directors of education were not willing to order schools to participate and thus, all forms of participation were known in advance. At the end of the recruiting process when the maximum number of intervention schools had been recruited, the rest of the interested schools were invited to participate as control schools. At least one control school was recruited from each participating municipality.

**Fig. 1:**
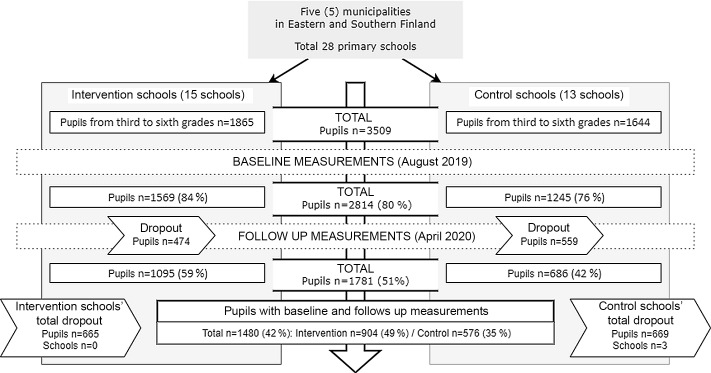
Study design, study population and measurements

Altogether fifteen intervention and thirteen control schools participated in the study (Fig. 1, three control schools withdrew). Each municipality had at least four participating schools, and all primary schools participated in one municipality. The number of pupils ranged from 51 to 424 (mean 192) in intervention schools and from 50 to 400 (mean 205) in control schools. In Finland, in 2019, the average number of pupils in a primary school was 169^(31)^.

### Measures

Pupils from grades 3–6 (aged 9–12 years) in the intervention and control schools completed a web-based study questionnaire in their classrooms with the support of a teacher two times, at baseline (August or September 2019, *n* 2814) and at the end of the school year (April 2020, *n* 1781). Pupils answered web-based questionnaires on the Formjack platform (version 3.1, Eduix Ltd, Finland 2008) during the school day with tablets or computers using personal response codes. The questionnaire contained items concerning eating patterns during school days, experiences of school dining, eating competence and body image.

Frequencies of eating school lunch were evaluated using a four-point scale: never, 1–2 times a week, 3–4 times a week, every school day, scored 1, 2, 3 and 4. Frequencies of eating different components of school meals according to the school meal recommendations^(22)^ warm main dish, salad or vegetable, salad dressing or oil, bread, margarine and drink (milk, buttermilk or a plant-based drink) were evaluated with the same scale as frequency of school lunch described above. In the current study, the balanced school meal was defined to contain a main dish, salad, bread, margarine and drink (milk, buttermilk, or a plant-based drink).

Experiences related to school dining were assessed using 16 statements (Table 1). These statements were evaluated using a five-point Likert scale: totally disagree, somewhat disagree, neither agree nor disagree, somewhat agree, totally agree, scored 1, 2, 3, 4 and 5. The statements were based on a previously published questionnaire^(32)^ that was developed further for the purposes of the current study by the study group. The questionnaire did not include a point-scale but instead contained individual statements. Nine statements were partly modified from the previously used questionnaire, and the other seven statements were developed by the research team.

**Table 1:** Pupils’ experiences related to school dining

Statement* (scores: min 1, max 5)	Committed intervention group *n* 536		Uncommitted intervention group *n* 367		Active control group *n* 243		Inactive control group *n* 334		*P* value†
The personnel are friendly									
Mean at baseline (sd)	4·36	0·87	4·10	0·93	4·43	0·76	4·37	0·90	
Mean at follow-up (sd)	4·35	0·82	4·08	1·05	4·35	0·86	4·30	0·86	0·665
It is peaceful in the dining hall									
Mean at baseline (sd)	2·91	1·16	2·43	1·22	2·92	1·11	2·80	1·11	
Mean at follow-up (sd)	2·99	1·10	2·63	1·08	2·94	1·05	2·99	1·13	0·183
There is no too much noise in the dining hall									
Mean at baseline (sd)	2·79	1·26	2·43	1·34	2·85	1·25	2·67	1·25	
Mean at follow-up (sd)	3·01	1·22	2·65	1·20	3·03	1·18	2·88	1·23	0·987
It is cozy in the dining hall									
Mean at baseline (sd)	3·82	1·16	3·33	1·24	3·90	1·08	3·67	1·20	
Mean at follow-up (sd)	3·89	1·02	3·36	1·19	3·80	1·12	3·67	1·13	0·441
The food queue runs smoothly									
Mean at baseline (sd)	3·66	1·13	3·54	1·22	3·63	1·19	3·84	1·13	
Mean at follow-up (sd)	3·65	1·09	3·57	1·07	3·71	1·02	3·88	1·08	0·841
School lunch is a nice moment in a day									
Mean at baseline (sd)	4·11	1·01	3·71	1·18	3·91	1·11	3·88	1·19	
Mean at follow-up (sd)	4·21	0·97	3·70	1·17	4·16	0·94	3·96	1·08	0·062
Teachers guide dining appropriately									
Mean at baseline (sd)	4·06	1·06	3·74	1·12	4·10	1·09	4·02	1·13	
Mean at follow-up (sd)	4·07	0·99	3·95	1·09^I^	4·03	1·15^I^	4·15	1·04^U,A^	0·013
I get to eat the amount I want at the school meal									
Mean at baseline (sd)	4·11	1·14	4·08	1·20	4·08	1·23	4·22	1·16	
Mean at follow-up (sd)	4·20	1·14^I^	4·11	1·13	4·14	1·22	4·02	1·25^C^	0·012
I have enough time to eat									
Mean at baseline (sd)	4·19	1·07	3·92	1·26	4·31	1·03	4·32	1·07	
Mean at follow-up (sd)	4·20	1·07	4·00	1·21	4·31	1·03	4·27	1·05	0·503
My table mates are very well behaved									
Mean at baseline (sd)	3·95	1·02	4·12	0·92	4·19	0·87	4·11	0·96	
Mean at follow-up (sd)	4·00	0·93	4·11	0·95	4·13	0·91	4·13	0·86	0·692
School meals are healthy									
Mean at baseline (sd)	4·32	0·93	3·99	1·02	4·19	0·91	4·16	0·98	
Mean at follow-up (sd)	4·35	0·87	4·07	1·00	4·34	0·86	4·19	1·02	0·421
School meals are tasty									
Mean at baseline (sd)	3·78	1·09	3·16	1·24	3·48	1·13	3·42	1·22	
Mean at follow-up (sd)	3·82	1·04	3·07	1·28	3·60	1·01	3·34	1·15	0·073
School meals look good									
Mean at baseline (sd)	3·69	1·11	3·11	1·26	3·53	1·09	3·27	1·23	
Mean at follow-up (sd)	3·64	1·06	2·99	1·24	3·57	1·04	3·28	1·13	0·291
School meals help me to stay healthy									
Mean at baseline (sd)	3·97	1·05	3·63	1·17	3·82	1·02	3·82	1·12	
Mean at follow-up (sd)	4·06	0·97	3·69	1·13	4·04	1·04	3·83	1·14	0·153
School meals help me to manage									
Mean at baseline (sd)	4·16	1·06	3·82	1·21	4·06	1·04	4·08	1·16	
Mean at follow-up (sd)	4·27	0·95^U,I^	3·92	1·15^C,A^	4·23	0·96^U^	4·01	1·14^C^	0·024
I get to participate in planning school dining									
Mean at baseline (sd)	2·41	1·30	2·41	1·35	2·29	1·27	2·53	1·39	
Mean at follow-up (sd)	2·67	1·28^A,I^	2·55	1·28	2·44	1·36^C^	2·46	1·31^C^	0·017

C = committed intervention group, U = uncommitted intervention group, A = active control group, I = inactive control group.

* Perspectives and experiences of school dining were evaluated using a 16-item query with a five-point Likert-scale: totally disagree, somewhat disagree, neither agree nor disagree, somewhat agree, totally agree. Questionnaire was edited from the study of Nutrition and Wellbeing of Secondary School Pupils^(28)^.

† *P* value of the interaction. The data were analysed with a mixed-effects model for repeated measures accounting for the intervention effect and selected standardising effects. The top index indicates which group the significance is given with.

Gender and class level were standardised in the analysis.

Eating competence was measured using a Finnish translation^(21)^ of the Satter Eating Competence Inventory (ecSI 2·0^TM^)^(33,34)^. The Satter Eating Competence Inventory is composed of four subscales (eating attitudes, contextual skills, food acceptance and internal regulation) comprising sixteen statements with a five-point Likert-scale: never, rarely, sometimes, often, always and scored 0, 0, 1, 2 and 3. A total score of at least thirty-two points indicates eating competence.

Body image was evaluated using three statements (I feel good about my body, I am attentive to my body’s needs and I am comfortable in my body) from a Finnish translation of the Body Appreciation Scale-2^(35,36)^. As the Body Appreciation Scale-2 for children^(37)^ was not available in Finnish. Thus, Body Appreciation Scale-2 was piloted with a small group of children and, based on the pilot, three questions were selected which best answered our research question. Body image was evaluated with a five-point Likert-scale: never, rarely, occasionally, often, always, scored 1, 2, 3, 4 and 5. Subjective experience of one’s own health status was evaluated by one question from the School Health Promotion Study^(38)^ with a modified five-point Likert scale: very poor, quite poor, moderate, quite good, very good, scored 1, 2, 3, 4 and 5.

#### Evaluation of the implementation strength

All principals of the intervention (*n* 15) and control schools (*n* 10) were individually interviewed to investigate the implementation strength of food education during the past school year. The interviews were conducted by telephone after the intervention in June or October 2020. Principals in the intervention schools were asked: (1) whether the Tasty School was included into the school year plan; (2) whether food education projects in which the entire school participated were implemented during the previous school year; (3) whether any positive changes were noticed at school (subjective evaluation) and (4) whether the school intended to take advantage of the Tasty School next school year. Principals in the control schools were asked: (1) whether food education projects in which the entire school participated were implemented during the previous school year and (2) whether the school took advantage of the Tasty School materials (freely available on the internet). In addition, principals were invited to give open feedback about the programme during the interview.

### Procedures

All intervention schools were supported to implement the Tasty School model during 1 school year from September 2019 to March 2020. Each intervention school selected 1–2 coordinating teachers who encouraged school personnel to implement food education in the school. Before the intervention, a researcher introduced the model to coordinating teachers. These coordinating teachers received a 1-d live training about the Tasty School at the beginning of the school year in September 2019. Researchers kept in contact with the coordinating teachers by email or telephone once a month throughout the school year.

For each intervention school, the starting point of the implementation was to complete a self-assessment questionnaire at the beginning of the school year concerning the state of their school’s food education and school lunch arrangements. The survey covered five themes: management and engagement, integration of food education, implementation of school meals, collaboration and support. The questionnaire was completed by a multi-professional group. Schools were instructed to invite at least a principal, a teacher and a food service employee into the multi-professional group, but others were also welcomed to participate. Based on the self-assessment questionnaire, each intervention school was advised to choose development targets to guide the implementation of the Tasty School at school level. The self-assessment questionnaire was used only for assessing the current state of food education and setting targets for schools. The information gathered from it was not used as research data.

Teachers in the intervention schools were instructed to utilise the Tasty School idea bank, which contained over 100 development or action ideas for food education in school. Each classroom teacher was instructed to put into practice at least one food education idea monthly (duration at least 30 min). This would mean at least seven ideas per school year (from September to March). Teachers were instructed to integrate these ideas into the school’s daily routines during the school year. The idea bank was freely available on the website (www.maistuvakoulu.fi, only in Finnish) and had three main sections: ideas for lessons, school dining and collaboration in food education. The ideas were grouped by topic for example, food culture, food systems, sensory-based learning (Sapere), media literacy, sustainable diets, body image and nutrition and health. Classroom teachers were also encouraged to complete during the autumn semester a 3-hour independent online training about food education that was freely available on the Tasty School website.

#### Supplemental intervention support during the school year

A researcher visited each intervention school two to three times during the intervention year. A monthly newsletter including practical ideas for food education was emailed to all the teachers in the intervention schools. Weekly food education tips were given through the Tasty School Facebook and Instagram pages. A monthly food education blog entry including topical issues and tips was published on the Tasty School website. Additionally, during the intervention year, one optional webinar was available for teachers and one for principals. The intervention schools received a printed copy of the Finnish Recommendations for School Meals and a toolkit for sensory-based food education activities. No financial support was given to any school.

### Data analysis

The principals’ interviews were analysed using qualitative content analysis. Data were first coded and then codes were organised into thematic statements. An intervention school was classified as a committed school if at least three statements were completed concerning implementation of the Tasty School food education model (see Table 2). A control school was classified as an active school if at least three statements in the principals’ interview were completed (see Table 3). To investigate the effects of the implementation strength of food education, schools were divided in the data analysis into four groups (committed intervention, uncommitted intervention, active control and inactive control) according to the strength of implementation reported in principals’ interviews.

**Table 2:** Description of planning and implementation of the tasty school at school level and whole school commitment to the intervention according to principal interviews

Statement	Intervention school														
	I1	I2	I3	I4	I5	I6	I7	I8	I9	I10	I11	I12	I13	I14	I15
Planning															
The self-assessment questionnaire was completed at baseline.	X	X	X	X	X	X	X	X	X		X	X	X	X	X
The self-assessment questionnaire was completed at follow-up.	X					X				X		X	X	X	X
Coordinating teacher(s) participated the 1-d live training.	X	X	X	X	X	X	X	X	X	X	X	X	X	X	X
The Tasty School was written into the school year plan.	X	X	X	X	X	X	X	X	X	X	X	X	X	X	X
The school also planned to use the Tasty School during the next school year.	X		X	X	X	X			X	X	X	X	X	X	X
Implementation															
The school staff co-operated with the food service staff.		X	X			X	X					X	X	X	X
The principal was active in promoting food education on the whole school level.						X	X		X	X		X	X	X	X
The school actively improved school dining.				X			X		X	X	X	X	X	X	X
The school organised a food educational project or workshop that involved all pupils.			X			X			X			X	X	X	X
Summary*															
The school was committed to the intervention.						X	X		X			X	X	X	X

* The school was classified as a committed school if at least three statements were completed concerning implementation of the model.

**Table 3:** Description of control schools’ food education activity according to principal interviews

Statement	Control school									
	C1	C2	C3	C4	C5	C6	C7	C8	C9	C10
The school staff co-operated with the food service staff.	X		X		X	X		X	X	
The principal was active in promoting food education on the whole school level.			X		X			X	X	
The school actively improved school dining.	X		X		X		X		X	
The school organised a food educational project or workshop that involved all pupils.			X		X	X		X	X	
Summary*										
The school was classified as active in food education.			X		X			X	X	

* The school was classified as an active school if at least three statements were completed. Three control schools did not participate in follow-up measurements and thus were not included.

Statistical analysis was performed using SPSS (IBM SPSS, version 27.0, 2020). The criterion for significance was set to be *P* < 0·05. Descriptive statistics (means, standard deviations and frequencies) were calculated separately for baseline and follow-up. Associations with frequencies of eating school lunch, perspective variables and explanatory variables were analysed using linear mixed-effects models for repeated measures in order to account for the multi-level data structure by clustering the repeated outcome measures at baseline and follow-up within pupils, who in turn, were clustered within schools. However, the clustering effect of school was found negligible when the activity of schools was considered. Thus, to avoid unnecessary complexity in the analysis, clustering effect of school was not included in the final model. The model was adjusted for gender and class level (grades 3–6), and included main effects for time and four-level intervention groups with intervention group × time interaction. Cross-tabulated frequencies between eating competence and the variable describing eating a balanced school meal were analysed with the *χ* ^2^-test.

#### Dropout analysis

Dropout analysis was conducted with the Mann–Whitney *U* test to examine possible selection bias between pupils (*n* 1329) who withdrew after the baseline and pupils (*n* 1480) who remained in the study. Few differences in perceived health status and food choices at school lunch were found. The mixed-model analysis was able to account for these differences.

A comparison of variable means showed that pupils who did not respond to the follow-up questionnaire experienced their health status as being significantly worse (means 1·7 and 4·30); in addition, they ate a main dish and salad at school meals less often than those pupils who answered both baseline and follow-up questionnaires. Nevertheless, there were no differences between the two groups in eating a balanced school meal every day, perspectives on school dining, eating competence total scores and perception of body image.

## Results

### Final study population and classification of participating schools

The final study sample consisted of 1480 pupils from grades 3 to 6 who had answered questionnaires both at baseline and at follow-up. Supplemental Table 1 displays descriptive information about participating pupils. In total, seven schools were classified as committed intervention, six schools as uncommitted intervention, four schools as active control and four schools as inactive control group (Tables 2 and 3). Descriptive information about study participants reveals that pupils in uncommitted intervention schools experienced their health status as being worse than in other schools reported (see online Supplemental Table 1).

### Pupils’ eating patterns and experiences related to school dining

#### Eating patterns at school meal

**Fig. 2:**
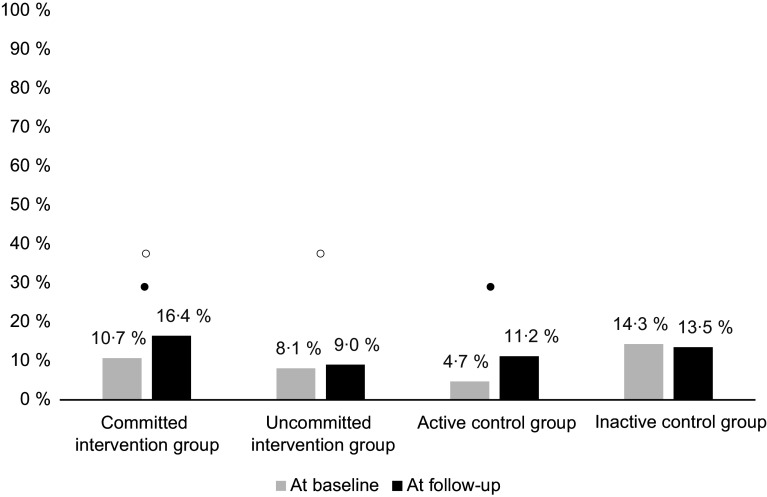
Pupils eating a balanced school meal daily at baseline and follow-up situations. The data were analysed with a mixed-effects model for repeated measures accounting for the intervention effect and selected standardising effects. *P* value of the interaction is 0·027. The significant differences in pairwise comparisons made with least-squares difference method are given between ○ Committed intervention group and Uncommitted intervention group, as well as ● Committed intervention group and Active control group. The balanced school meal contains main dish, salad, bread, margarine and drink (milk, buttermilk or a plant-based drink)

At baseline, 88 % of pupils ate school lunch every school day, but only 10 % of pupils ate a balanced school meal every school day, including main dish, salad, bread with margarine and drink (milk, buttermilk or a plant-based drink). Consumption of a balanced school meal increased in the committed (from 10·7 % to 16·4 %) and uncommitted (from 8·1 % to 9·0 %) intervention groups as well as in the active control group (from 4·7 % to 11·2 %) during the school year (*P* = 0·027, Fig. 2). In the inactive control group, the consumption of a balanced school meal decreased. The change was greater among pupils in the committed intervention and active control groups than in the uncommitted intervention group.

#### Experiences related to school dining

Pupils’ experiences of school dining at baseline and follow-up are presented in Table 1. At baseline, 45 % of pupils expressed that there is not too much noise in the dining hall, 62 % that the dining hall is cozy, 70 % that the school meal is a nice moment in the day, 78 % that they have enough time to eat and 56 % that school meals are tasty. In the follow-up pupils, especially in the committed intervention group, experienced that their opportunities to participate in school dining planning increased, and pupils in the inactive control group felt their opportunities decreased (*P* = 0·017). Pupils’ opportunities to eat the amount of food they desired at the school meal increased in both committed and uncommitted intervention groups (*P* = 0·012). At follow-up situation, the pupils in committed intervention, uncommitted intervention and active control groups were more likely to report that the school meal helps them to manage, whereas in the inactive control group this experience decreased (*P* = 0·024). Teachers’ appropriate guidance at the school meal increased in the inactive control group (*P* = 0·013).

#### Eating competence

46 % of all pupils were classified as competent eaters (girls 47 %, boys 44 %), and the eating competence mean total score was 30·0 at baseline. Eating competence was associated with eating more commonly a balanced school meal (compare 5·5 % to 15·0 %, *P* = 0·001). Eating competence total score increased (*P* = 0·025, see online Supplemental Fig. 1) in the committed intervention group (baseline 29·4 and at follow-up 31·5). The increase was found specifically in food acceptance subscore (*P* = 0·001), which increased significantly in all the groups, except in the uncommitted intervention group (*P* = 0·001, see online Supplemental Table 2).

#### Perception of body image

A total of 41 % of pupils (girls 35 %, boys 47 %) always felt good and 35 % often felt good (girls 35 %, boys 35 %) about their bodies. A total of 9 % of pupils (girls 12 %, boys 6 %) never or rarely felt good about their bodies. No significant changes were found in perception of body image during intervention or between study groups (see online Supplemental Table 3).

## Discussion

This study found that only a minority of pupils (10 %) consumed all components of school meals as recommended by the Finnish National Nutrition Council^(22)^. Thus, the current study strengthened earlier study findings that balanced school meals are consumed rarely^(13)^, although they are offered to all pupils free of charge. The current study also found that active implementation of food education in the school environment can improve pupils’ eating patterns—eating a balanced school meal, even without making any actual changes to the composition of the school meal, as previously reported^(17)^. In practice, school personnel selected actions they felt were needed and many schools changed the way the salad was offered to be more attractive, encouraged tasting and enhanced the atmosphere of the dining hall by lowering noise with weekly quiet dining.

The main aim of the Tasty School model was to offer primary schools an inclusive model for food education, which has shown to be an effective strategy^(39)^. According to the results, the model achieved this goal. The previous study showed that according to teachers’ and principals’ experience, the Tasty School model is feasible for food education in primary schools^(24)^. The current study adds to our understanding of the effects of Tasty School on pupils, i.e. whether the model achieves its intended outcomes. The pupils in the committed intervention school experienced higher social participation in planning school dining than pupils in control school. In practice, teachers in the intervention schools created common rules for school dining together with pupils and organised workshops where pupils carried out SWOT (strengths, weaknesses, opportunities, threats) analysis for school dining.

In this study, 46 % of pupils were classified as eating competent. The amount is slightly lower than in an earlier study in which 58 % of pupils were classified as eating competent^(21)^. However, it should be noted that in this study, subjects were younger than in the previous study. More information is needed on the development of eating competence with age. In an earlier study involving adults, eating competence was more common among older subjects^(40)^. Interestingly, eating competence was statistically associated with eating a balanced school meal. The finding reinforces the earlier findings^(21)^ that the thematic areas of the ecSatter model could be important targets for health promotion. Eating competence did not increase after the intervention except in food acceptance, one of the eating competence subscores. In the present study, the use of Sapere^(27)^ sensory-based education activities may be one reason for increased food acceptance among the pupils in the committed intervention school. Sapere is an essential part of the Tasty School model. In practice, by using Sapere activities, pupils were offered an opportunity to explore vegetables and other foods by smelling, tasting, touching, seeing and hearing. Earlier interventions using the Sapere method in nutrition education have also been successful in reducing food neophobia, encouraging school-aged children to try new, unfamiliar foods and expand their food repertoire^(27,41,42)^.

Traditionally food and nutrition education at school has not focused on pupils’ body image^(43)^. Themes related to body image were included in the Tasty School model, as body image dissatisfaction affects eating patterns negatively^(44)^. At baseline, fewer than half (41 %) of pupils always felt good about their bodies, and 9 % of pupils never or rarely felt good about their bodies. Similar results were found in Spain, where 43 % of pupils of the same age (9–12 years old) were satisfied with their bodies^(45)^. In future research, an adapted version of the questionnaire Body Appreciation Scale-2 for children (BAS-2C)^(37)^ could be a useful measure to get more specific information on children’s body image.

No changes in perception of body image were observed during the intervention in the current study. The result is similar to the findings of our previous study among fifth grade pupils^(17)^. In line with the principles of the Health at Every Size approach^(26)^, this can also be regarded as a positive result, indicating that implementing the Tasty School model did not cause harm to pupils’ body image. The results of the current study suggest that there might be gender differences in body image of children. Moreover, body image is constructed of multi-component factors and strongly affected by, for example, media^(46)^ and social media^(47)^. It could thus be assumed that changes in perception of body image require a more intensive or longer period of intervention. The development of a balanced body and food relationship for children and adolescents needs support from adults, and it is therefore important to consider the manner in which food education is delivered.

The implementation strength of food education influenced the observed changes in pupils’ eating patterns and experiences related to school dining. Our findings suggest that commitment at the whole school level is important, and school administrators could increase the effectiveness of the actions implemented by teachers to promote food education. The crucial role of school administrators or principals has also been demonstrated in previous studies from South Korea and China, which concluded that a school administrator’s lack of priority for nutrition education or an administrator not requiring teachers to teach nutrition is a barrier to implementation^(48,49)^.

The current study demonstrated that in addition to evaluating results of individual food education actions, it is also important to evaluate implementation. Approximately half of the intervention schools were classified as uncommitted intervention schools. These schools were not able to implement Tasty School as expected. The results of the current study do not reveal reasons for unsuccessful implementation. However, the self-assessed health status of pupils was worse in uncommitted intervention schools. This might have affected the whole school culture and routines, challenging teachers’ work and limiting their resources for further developmental work. Finally, almost half of the control schools were classified as active control schools based on the post-intervention interviews of school principals. In Finland, food education is included in the Finnish Basic Education Curriculum^(16)^, and thus we could not prevent control schools from applying active food education as part of their school culture. It was thus encouraging to observe that any activities related to food education were accompanied with favourable outcomes.

### Strengths and limitations

A strength of the current study was the large number of study subjects^(50)^. The research data were also collected extensively across Finland; thus, the results are not limited to a particular area. Furthermore, the schools varied in size and operated in several municipalities, so the results are not the result of an individual municipality’s resources or curriculum. Another strength was that the intervention was carried out as part of normal schoolwork (e.g. conducted by teachers, not researchers), improving the applicability of the results to practice.

A further strength of this study was that the interpretation of the results considered the intensity of the implementation of food education and thus was able to compare schools with respect to implemented food education. The data were collected using internet-based questionnaires that the pupils completed on schooldays. In this way, the study did not select participants and the data can be seen as descriptive. However, data were collected using self-reported questionnaires instead of objective measures to assess eating patterns.

The study also has several limitations. As a real-life quasi-experimental study, this intervention may have been subject to selection bias. Participating schools self-selected to participate either as an intervention or control school and thus schools could not be randomised into research groups. However, the schools were divided into four groups according to strength of food education implementation, which decreased the possibility of bias in the results. To confirm our findings, future studies should use randomised study designs and intention to treat analyses.

The intervention study was conducted in the 2019–2020 school year, during which the COVID-19 pandemic emerged and forced schools to shift into remote teaching 1 month before the follow-up measurements. Although pupils were instructed to think of an ordinary school day at school when answering the follow-up questionnaire, we cannot rule out the potential effects of this unusual situation. However, the situation was the same in all participating schools, and thus the intervention–control study design likely alleviated these possible effects. Instead, the COVID-19 pandemic might not have greatly impacted teachers’ resources and thus the implementation strength of food education because the pandemic was present only during the last month of the intervention.

On the other hand, the COVID-19 pandemic might have affected the dropout rates. Lower dropout rates in intervention groups indicated their higher commitment to the study. However, the baseline dropout analysis found no significant differences between the pupils who withdrew compared to those who completed the study. Finally, as a quasi-experimental study, the study frame might be exposed to selection bias.

The observed changes in eating patterns were, though encouraging, rather small. The duration of the current study was one school year, and it could be expected that with a longer duration of food education, stronger effects could be seen. Additionally, the holistic approach to food education should be more acknowledged and promoted^(43)^ and should also be considered in the education of future teachers.

## Conclusions

Commitment at the school level and active implementation of food education had beneficial effects on pupils’ eating patterns and experiences of school dining. The Tasty School food education model offers an effective tool to primary schools for promoting healthy eating patterns and social participation in school dining among pupils not only in Finland, but also potentially in other countries as well.

The current study indicates that whole school commitment and the activity of food education are crucial for beneficial outcomes on pupils’ eating patterns. School administrators and curricula must encourage planning and implementing food education activities and practices through the whole school year to ensure the effective strength of the implementation of food education.

## Supplementary material

For supplementary material accompanying this paper visit https://doi.org/10.1017/S1368980022002154. click here to view supplementary material
