# Supplementary material for: Implementation of food education in school environments improves pupils’ eating patterns and social participation in school dining
Source: Public Health Nutr. 2022 Oct 10;25(12):3548–58. doi: 10.1017/S1368980022002154 (PMC9991779; doi:10.1017/S1368980022002154)
Supplement: Supplementary file 1 [file S1368980022002154sup001.docx]

●

●

○

○

Supplementary Figure 1. **Eating Competence Total Score Among Pupils at Baseline and Follow-up**

The data were analyzed with a mixed-effects model for repeated measures accounting for the intervention effect and selected standardizing effects. *P* value of the interaction is 0.025. The significant differences in pairwise comparisons made with least-squares difference method are given between ○ Uncommitted intervention group and Committed intervention group, as well as ● Uncommitted intervention group and Inactive control group. Cutoff point for being a competent eater is 32 points or more (min 0, max 48).
